# Late morbidity and mortality after autologous blood or marrow transplantation for lymphoma in children, adolescents and young adults—a BMTSS report

**DOI:** 10.1038/s41375-024-02144-7

**Published:** 2024-02-19

**Authors:** Anna Sällfors Holmqvist, Qingrui Meng, Chen Dai, Lindsey Hageman, Wendy Landier, Jessica Wu, Liton F. Francisco, Elizabeth Schlichting Ross, Nora Balas, Alysia Bosworth, Hok Sreng Te, Ravi Bhatia, Joseph Rosenthal, F. Lennie Wong, Daniel Weisdorf, Saro H. Armenian, Smita Bhatia

**Affiliations:** 1 Childhood Cancer Center, Skåne University Hospital, Department of Clinical Sciences, Lund University, Lund, Sweden; 2 Institute for Cancer Outcomes and Survivorship, University of Alabama at Birmingham School of Medicine, Birmingham, AL USA; 3 Population Sciences, City of Hope, Duarte, CA USA; 4 Division of Hematology, Oncology and Transplantation, University of Minnesota, Minneapolis, MN USA; 5 Division of Hematology, Oncology and Bone Marrow Transplantation, University of Alabama at Birmingham, Birmingham, AL USA; 6 Pediatric Hematology/Oncology, City of Hope, Duarte, CA USA; 7 Division of Pediatric Hematology, Oncology and Bone Marrow Transplantation, University of Alabama at Birmingham, Birmingham, AL USA

**Keywords:** Cancer epidemiology, Epidemiology

## Abstract

We determined the risk of late morbidity and mortality after autologous blood or marrow transplantation (BMT) for lymphoma performed before age 40. The cohort included autologous BMT recipients who had survived ≥2 years after transplantation (*N* = 583 [HL = 59.9%; NHL = 40.1%]) and a comparison cohort (*N* = 1070). Participants self-reported sociodemographics and chronic health conditions. A severity score (grade 3 [severe], 4 [life threatening] or 5 [fatal]) was assigned to the conditions using CTCAE v5.0. Logistic regression estimated the odds of grade 3–4 conditions in survivors vs. comparison subjects. Proportional subdistribution hazards models identified predictors of grade 3–5 conditions among BMT recipients. Median age at BMT was 30.0 years (range: 2.0–40.0) and median follow-up was 9.8 years (2.0–32.1). Survivors were at a 3-fold higher adjusted odds for grade 3–4 conditions (95% CI = 2.3–4.1) vs. comparison subjects. Factors associated with grade 3–5 conditions among BMT recipients included age at BMT (>30 years: adjusted hazard ratio [aHR] = 2.31; 95% CI = 1.27–4.19; reference: ≤21 years), pre-BMT radiation (aHR = 1.52; 95% CI = 1.13–2.03; reference: non-irradiated), and year of BMT (≥2000: aHR = 0.54; 95% CI = 0.34–0.85; reference: <1990). The 25 years cumulative incidence of relapse-related and non-relapse-related mortality was 18.2% and 25.9%, respectively. The high risk for late morbidity and mortality after autologous BMT for lymphoma performed at age <40 calls for long-term anticipatory risk-based follow-up.

## Introduction

High-dose chemotherapy ± radiation with autologous blood or marrow transplantation (BMT) rescue is an integral part of consolidation and/or salvage therapy for children, adolescents and young adults with Hodgkin lymphoma (HL) and non-Hodgkin lymphoma (NHL) [1, 2]. While conventionally treated cancer patients are at risk for late morbidity and mortality [3–7], the high-intensity conditioning increases the risk further [8]. However, there is a paucity of information regarding the long-term burden of morbidity and cause-specific late mortality after autologous BMT for lymphoma performed in children, adolescents and young adults. The few studies that have examined this question are limited by small samples or relatively short follow-up [9–11]. We addressed these limitations by leveraging the Blood or Marrow Transplant Survivor Study (BMTSS) to determine the magnitude of risk of chronic health conditions as well as cause-specific late mortality in patients undergoing autologous BMT for lymphoma before age 40. We also sought to determine sub-populations at highest risk for morbidity to set the stage for future targeted interventions.

## Methods

### Study design and participants

BMTSS is a cohort study examining long-term outcomes in individuals who received BMT between 1974 and 2014 at the University of Minnesota, City of Hope, or University of Alabama at Birmingham and survived 2 or more years after BMT, regardless of disease status at cohort entry or vital status after cohort entry. A comparison cohort of siblings of all the BMT survivors participating in the BMTSS was also enrolled. However, only 16% of the BMT survivors were related to the siblings included in this analysis. Survivors and comparison subjects (or parents of participants <18 years) completed a 255-item BMTSS survey covering the following areas: diagnosis by a healthcare provider of chronic health conditions (including age at diagnosis), healthcare utilization and sociodemographic characteristics (race/ethnicity, sex, education, household income and insurance). The reliability and validity of the BMTSS survey was tested, and responses regarding chronic health conditions demonstrated a high level of agreement with the medical records [12]. Chronic health conditions were graded using the Common Terminology Criteria for Adverse Events (CTCAE), v5.0, distinguishing grades 1 through 5 based on the severity of each event (grade 1, mild; grade 2, moderate; grade 3, severe; grade 4, life-threatening/disabling; grade 5, death from chronic health conditions) [13]. For participants with more than one chronic health condition, the condition with a maximum grade was used. The same scoring system was applied to responses from the comparison cohort. A detailed description of the questions asked in the BMTSS survey, the corresponding chronic health condition categories created from the responses and the scoring of these conditions are presented in Supplementary Table 1. Information on primary diagnosis, age at BMT, stem cell source (bone marrow; peripheral blood stem cells [PBSCs]), risk of relapse at transplantation (high or standard risk), and exposure to total body irradiation (TBI) and chemotherapeutic agents used for conditioning, as well as pre-BMT therapeutic exposures was obtained on BMT recipients from the institutional transplant databases and/or medical records. Patients who underwent BMT in first or second complete remission were considered standard risk of relapse at transplantation; all other patients were considered at high risk. To be included in the current report, BMTSS participants with HL or NHL had to have received a single or tandem (two transplantations within 3 months) autologous BMT before age 40.

National Death Index (NDI) Plus [14] provided data regarding the date and cause of death through December 31, 2020. Additional information from medical records and Accurint databases [15] were used to extend the vital status information through May 31, 2022. For the deceased patients, we used the cause of death information from the NDI Plus; a CTCAE grade 5 was assigned to chronic health conditions stated as the cause of death in the NDI Plus files for those who died of a chronic health condition. Cause of death was classified as external (suicide, homicide or accident), relapse-related (RRM: primary cause of death matching the pre-transplant diagnosis) and non-relapse-related (NRM). The University of Alabama at Birmingham institutional review board served as the single institutional review board of record. Informed consent was obtained in accordance with the Declaration of Helsinki.

### Statistical analyses

#### BMT survivors (alive at study participation) vs. comparison cohort

Standard parametric and nonparametric techniques compared the demographic and clinical characteristics and the prevalence of specific chronic health conditions between BMT survivors and the comparison cohort. Cumulative incidence of chronic health conditions as a function of attained age compared the burden of morbidity between BMT recipients and comparison subjects. Logistic regression was used for estimating the odds of grade 3–4 conditions in BMT survivors vs. the comparison cohort, adjusting for age at study participation, sex, race/ethnicity, education, household income and health insurance.

#### BMT cohort (alive and deceased)

For the BMT cohort, the cumulative incidence of chronic health conditions was calculated as a function of time from BMT, using death from other causes as competing risk. When analyzing predictors of grade 3–5 conditions in BMT recipients, proportional subdistribution hazards models (Fine-Gray) were used, treating death from primary disease or external causes as competing risk. The following variables were examined: sex, race/ethnicity, age at BMT, year of BMT, primary disease, source of stem cells, pre-BMT therapeutic exposures, TBI, chemotherapeutic agents used for conditioning, treating institution, education, household income and health insurance. BMT recipients with missing pre-BMT treatment data were treated as a separate group. For the multivariable model, we performed stepwise backward variable selection, retaining variables with *p* values < 0.1 in the final models. Mediation analysis was done by adding individual risk factors to the model examining the association between transplant era (<1990; 1990–1999; ≥2000) and risk of grade 3–5 conditions, censored at 7 years post-BMT, to examine if the hazard ratios changed. Censoring at 7 years post-BMT was chosen to ensure comparable follow-up across all eras. Overall survival was calculated using the Kaplan–Meier method, conditional on surviving 2 years post-BMT. The cumulative incidence of RRM used NRM and external causes as competing risk. The cumulative incidence of NRM used RRM and external causes as competing risk.

Data were analyzed using SAS version 9.4 (SAS Institute, Cary, NC). All statistical tests were two-sided, and *p* < 0.05 was considered statistically significant.

## Results

Of the 862 BMT recipients eligible for study participation, 223 (25.9%) had died after surviving at least 2 years (Supplementary Fig. 1). Of the 639 alive eligible BMT survivors, 122 were lost to follow-up (19.1%). Of the 517 patients successfully contacted, 157 patients refused participation (30.4%), yielding 360 participants who completed the BMTSS questionnaire (participation rate: 69.6%). The cohort used in this report consisted of 583 individuals (360 alive and 223 deceased) who had received autologous BMT for HL or NHL before 2014 at age <40 years, and had survived ≥2 years. Females (46.7% vs. 31.8%, *p* = 0.001), non-Hispanic White individuals (72.7% vs. 62.4%, *p* = 0.007) and those transplanted before year 2000 (56.1% vs. 24.2%, *p* < 0.001), were more likely to participate (Supplementary Table 2).

### Study cohort

The characteristics of the BMT recipients overall and by primary disease are shown in Table 1. In this cohort of 583 patients, 349 (59.9%) carried a primary diagnosis of HL and 234 (40.1%) of NHL. The median age at transplantation was 30.0 years (range: 2.0–40.0); median length of follow-up was 9.8 years (2.0–32.1); median age at study participation was 41.0 years (13.7–67.1); 311 were male (53.3%), 424 were non-Hispanic White (72.9%), and 255 had undergone BMT in the year 2000 or later (43.7%). PBSCs were used in 493 individuals (84.6%), and 367 were considered at high risk of relapse at BMT (63.0%). TBI was used for conditioning in 222 individuals (38.1%) (median dose: 12.0 Gy, range 5.0–13.2 Gy). Of the 438 (75.1%) individuals with available data on pre-BMT therapeutic exposures, 166 (37.9%) had received pre-BMT radiation with a median radiation dose of 32.5 Gy (range 10.0–60.0 Gy). The comparison cohort consisted of 1070 individuals. A comparison of the demographic characteristics between comparison subjects and BMT survivors alive at study participation is shown in Supplementary Table 3.

**Table 1:** Demographic and clinical characteristics of 2-year survivors of autologous BMT for lymphoma performed before age 40 years.

	All patients *N* = 583	Hodgkin lymphoma *N* = 349 (59.9%)	Non-Hodgkin lymphoma *N* = 234 (40.1%)	*p* value
Age at BMT in years				
Median (range)	30.0 (2.0–40.0)	28.4 (7.2–40.0)	32.0 (2.0–40.0)	<0.0001
Follow-up in years				
Median (range)	9.8 (2.0–32.1)	8.0 (2.0–32.1)	12.2 (2.2–31.8)	<0.0001
Age at study participation/deceased				
Median (range)	41.0 (13.7–67.1)	38.3 (16.0–63.7)	44.3 (13.7–67.1)	<0.0001
Age at BMT in years, *N* (%)				
≤21	84 (14.4%)	63 (18.1%)	21 (9.0%)	0.0004
22–30	208 (35.7%)	133 (38.1%)	75 (32.1%)	
>30	291 (49.9%)	153 (43.8%)	138 (59.0%)	
Sex, *N* (%)				
Female	272 (46.7%)	169 (48.4%)	103 (44.0%)	0.3
Race/ethnicity, *N* (%)				
Non-Hispanic white	424 (72.9%)	258 (73.9%)	166 (70.9%)	0.5
Black	38 (6.5%)	23 (6.6%)	15 (6.4%)	
Hispanic	86 (14.8%)	51 (14.6%)	35 (15.0%)	
Other	35 (6.0%)	17 (4.9%)	18 (7.7%)	
Year of BMT, *N* (%)				
<1990	76 (13.0%)	44 (12.6%)	32 (13.7%)	0.9
1990–1999	252 (43.2%)	152 (43.6%)	100 (42.7%)	
≥2000	255 (43.7%)	153 (43.8%)	102 (43.6%)	
Stem cell source, *N* (%)				
Bone marrow	90 (15.4%)	52 (14.9%)	38 (16.2%)	0.7
Peripheral blood stem cells	493 (84.6%)	297 (85.1%)	196 (83.8%)	
Total body irradiation, *N* (%)				
Yes	222 (38.1%)	71 (20.3%)	151 (64.5%)	<0.0001
Missing information	1 (0.2%)	1 (0.3%)	0 (0%)	
Disease status at BMT, *N* (%)				
Standard risk^b^	142 (24.4%)	63 (18.1%)	79 (33.8%)	<0.0001
High risk^b^	367 (63.0%)	236 (67.6%)	131 (56.0%)	
Missing information	74 (12.7%)	50 (14.3%)	24 (10.3%)	
Number of BMTs, *N* (%)				
1	537 (92.1%)	304 (87.1%)	233 (99.6%)	<0.0001
2	46 (7.9%)	45 (12.9%)	1 (0.4%)	
Treating institution, *N* (%)				
City of Hope	375 (64.3%)	208 (59.8%)	167 (71.1%)	0.003
UMN	143 (24.5%)	90 (25.9%)	53 (22.6%)	
UAB	65 (11.1%)	50 (14.4%)	15 (6.4%)	
Pre-BMT treatment, *N* (%)				
Information available	438 (75.1%)	252 (72.2%)	186 (79.5%)	0.05
Alkylators	436 (99.5%^a^)	251 (99.6%^a^)	185 (99.5%^a^)	0.8
Anthracyclines	431 (98.4%^a^)	248 (98.4%^a^)	183 (98.4%^a^)	1.0
Antimetabolites	194 (44.3%^a^)	82 (32.5%^a^)	112 (60.2%^a^)	<0.0001
Bleomycin	278 (63.5%^a^)	240 (95.2%^a^)	38 (20.4%^a^)	<0.0001
Monoclonal antibodies	80 (18.3%^a^)	14 (5.6%^a^)	66 (35.5%^a^)	<0.0001
Platinum	222 (50.7%^a^)	133 (52.8%^a^)	89 (47.8%^a^)	0.3
Topoisomerase inhibitors	249 (56.8%^a^)	151 (59.9%^a^)	98 (52.7%^a^)	0.13
Radiotherapy	166 (37.9%^a^)	118 (46.8%^a^)	48 (25.8%^a^)	<0.0001
Radiotherapy dose, Median (range)	32.0 (10.0–60.0)	35.5 (15.3–50.4)	30.0 (10.0–60.0)	0.4
Conditioning chemotherapy, *N* (%)				
Etoposide	525 (90.1%)	339 (97.1%)	186 (79.5%)	<0.0001
Nitrosourea	303 (52.0%)	233 (66.8%)	70 (29.9%)	<0.0001
Carmustine	297 (50.9%)	228 (65.3%)	69 (29.5%)	<0.0001
Deceased at study participation, *N* (%)				
	223 (38.3%)	162 (46.4%)	61 (26.1%)	<0.0001

*UMN* University of Minnesota, *UAB* University of Alabama at Birmingham.

^a^Percentage of BMT recipients with information on pre-BMT treatment available.

^b^Patients who underwent BMT in first or second complete remission were considered standard risk of relapse at transplantation; all other patients were considered at high risk.

### Severe/life-threatening chronic health conditions in autologous BMT survivors (alive at study participation) vs. comparison subjects

**Fig. 1:**
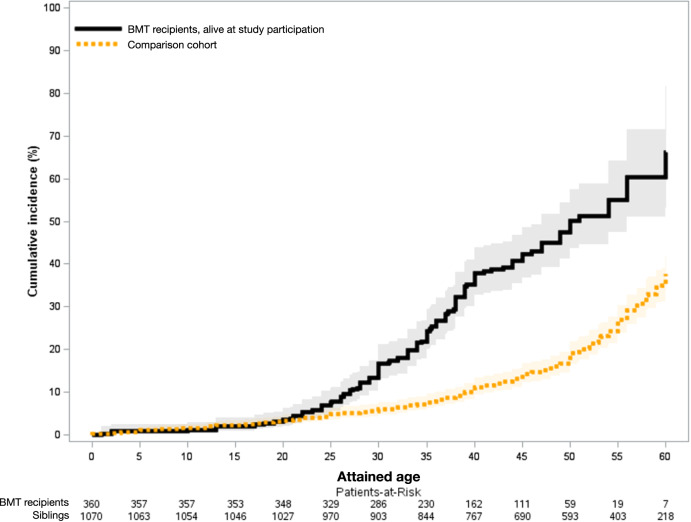
Cumulative incidence of grade 3–4 chronic health conditions among 2-year survivors of autologous BMT for lymphoma and comparison subjects, respectively, by attained age.

The prevalence of grade 3–4 chronic health conditions was significantly greater in survivors compared to comparison subjects (any condition: 39.3% vs. 26.8%, *p* < 0.0001; multiple conditions: 14.7% vs. 7.9%, *p* < 0.0001). By age 45, the cumulative incidence of a grade 3–4 chronic health conditions was 41.8% (95% CI; 35.8%–47.6%) among BMT survivors vs. 14.3% (95% CI; 12.1%–16.7%) among comparison subjects (*p* < 0.0001) (Fig. 1). The adjusted odds for grade 3–4 chronic health conditions was three-fold higher in BMT survivors compared to the comparison cohort (aOR = 3.0, 95% CI = 2.3–4.1) (Supplementary Table 4).

### Severe/life-threatening/fatal chronic health conditions among autologous BMT recipients

**Fig. 2:**
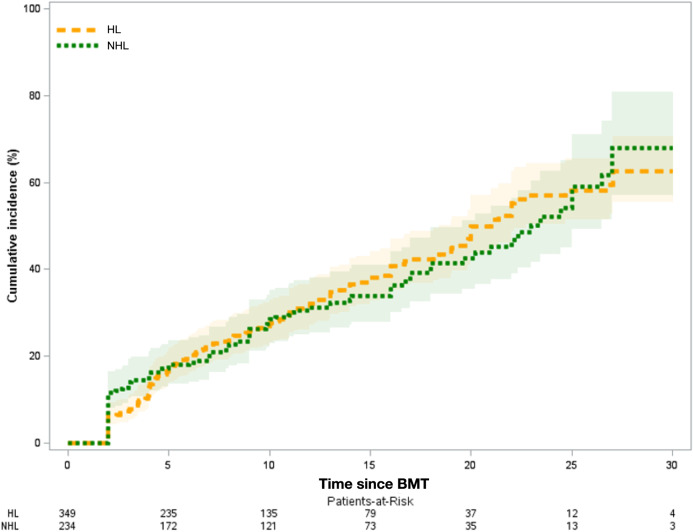
Cumulative incidence of grade 3–5 chronic health conditions among 2-year survivors of autologous BMT for lymphoma, by time since BMT.

Of the 583 BMT recipients in the cohort, 408 (70.0%) developed ≥1 grade 1–5 chronic health conditions (HL: *N* = 234 [67.1%]; NHL: *n* = 174 [74.4%]). Furthermore, 241 BMT recipients (41.3%) developed one or more grade 3–5 conditions (HL: *N* = 148 [42.4%]; NHL: *N* = 93 [39.7%]). The 20 years cumulative incidence of any grade 3–5 condition for the entire cohort was 47.1% (95% CI = 42.0–52.1%); it was 49.9% (95% CI = 43.2%–56.3%) for HL patients and 42.6% (95% CI = 34.7%–50.2%) for NHL patients (Fig. 2).

As shown in Table 2, the risk of a grade 3–5 condition in the entire cohort was higher among those transplanted at age >30 years (HR = 2.31; 95% CI = 1.27–4.19; reference: ≤21 years), and those receiving pre-BMT radiotherapy (HR = 1.52; 95% CI = 1.13–2.03; reference: non-irradiated). Patients treated in the most recent era (2000 or later) had a lower risk for a grade 3–5 condition compared to those transplanted before 1990 (HR = 0.54; 95% CI = 0.34–0.85). When restricting the follow-up to 7 years post-BMT, the lower risk among those transplanted in year 2000 or later remained significant (HR = 0.44; 95% CI = 0.26–0.74) (Supplementary Fig. 2). Among patients with HL, the risk of a grade 3–5 condition was higher among those transplanted at age >30 years (HR = 2.16; 95% CI = 1.12–4.18; reference: ≤21 years) (Table 2). Among patients with NHL, Black patients had a higher risk of a grade 3–5 condition (HR = 3.79; 95% CI = 1.88–7.67), as did those receiving pre-BMT radiotherapy (HR = 1.77; 95% CI = 1.13–2.76; reference: non-irradiated). Finally, patients with NHL transplanted ≥2000 had a HR of 0.34 (95% CI = 0.18–0.64) of a grade 3–5 condition compared to those transplanted before 1990. When restricting the follow-up to 7 years post-BMT, the lower risk among those transplanted in year 2000 or later remained significant among NHL patients (HR = 0.33; 95% CI = 0.14–0.75). In the mediation analysis, none of the measured risk factors explained the decrease in risk seen among those transplanted in the most recent era (Supplementary Table 5).

**Table 2:** Hazard ratio of any grade 3–5 chronic health condition for 583 2-year survivors of autologous BMT for lymphoma performed before age 40 years.

	All BMT recipients			Hodgkin lymphoma			Non-Hodgkin lymphoma		
Variable	HR	95% CI	*p* value	HR	95% CI	*p* value	HR	95% CI	*p* value
Age at BMT (years)									
≤21	Ref			Ref					
22–30	1.46	0.79–2.72	0.23	1.22	0.61–2.43	0.58			
>30	2.31	1.27–4.19	0.01	2.16	1.12–4.18	0.02			
Race/ethnicity									
Non-Hispanic white				Ref			Ref		
Black				0.53	0.24–1.14	0.10	3.79	1.88–7.67	0.0002
Hispanic				1.59	0.91–2.79	0.11	1.50	0.88–2.56	0.14
Other				1.25	0.58–2.66	0.57	0.75	0.25–2.29	0.61
Year of BMT									
<1990	Ref						Ref		
1990–1999	0.73	0.48–1.09	0.13				0.58	0.32–1.06	0.08
≥2000	0.54	0.34–0.85	0.01				0.34	0.18–0.64	0.001
Stem cell source									
Bone marrow				Ref					
PBSC				0.66	0.42–1.05	0.08			
Pre-BMT radiotherapy									
No	Ref			Ref			Ref		
Yes	1.52	1.13–2.03	0.01	1.47	1.00–2.18	0.05	1.77	1.13–2.76	0.01

The most prevalent grade 3–5 chronic health conditions included subsequent malignant neoplasms (11.6%), and cardiovascular disease (10.5%) (Supplementary Table 6). Specific subsequent malignant neoplasms included leukemia/MDS (23.9%), skin cancer (17.9%), breast cancer (11.9%), sarcoma (7.5%), gastro-intestinal cancer (6.0%), lung cancer (6.0%), other cancer types (16.4%) and unknown (10.4%). The prevalence of grade 3–5 cardiovascular disease among Black BMT recipients was 18.4% and among non-Hispanic white 9.7%. The 10 years cumulative incidence of grade 3–5 cardiovascular disease was 6.1% (95% CI = 4.2–8.5%) and of grade 3–5 subsequent malignant neoplasms was 5.6% (95% CI = 3.8–7.8%) (Supplementary Table 7).

### Late mortality in autologous BMT recipients

**Fig. 3:**
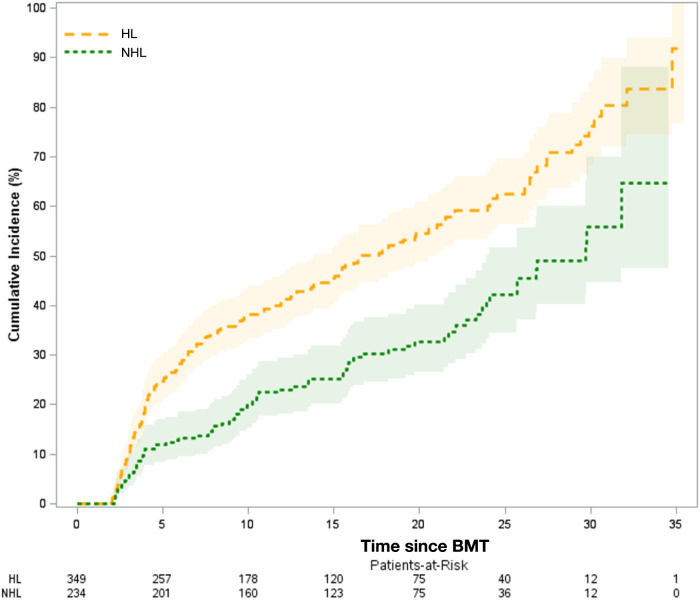
Cumulative incidence of all-cause late mortality among 2-year survivors of autologous BMT for Hodgkin lymphoma and non-Hodgkin lymphoma, respectively, performed before age 40 years.

Conditional on surviving the first 2 years after BMT, the 25 years all-cause mortality was 62.6% (95% CI = 55.5–68.8%) for HL patients and 42.3% (95% CI = 33.7–50.6%) for NHL patients (Fig. 3). The adjusted hazards of all-cause mortality were higher in individuals with HL (HR = 1.75, 95% CI = 1.20–2.55; reference: NHL), among Black BMT recipients (HR = 2.36, 95% CI = 1.42–3.90; reference: non-Hispanic White), and in those with a lower income ($50,000–$74,999: HR = 3.07, 95% CI = 1.39–6.79; <$50,000: HR = 5.12, 95% CI = 2.55–10.28; reference: ≥$75,000) (Table 3).

**Table 3:** Hazard ratio of all-cause late mortality among 2-year survivors of autologous BMT for lymphoma performed before age 40 years.

Variable	HR	95% CI	*p* value
Race/ethnicity			
Non-Hispanic white	Ref		
Black	2.36	1.42–3.90	0.001
Hispanic	1.41	0.89–2.23	0.14
Other	1.36	0.65–2.83	0.42
Year of BMT			
<1990	Ref		
1990–1999	1.58	0.92–2.72	0.10
≥2000	1.16	0.63–2.12	0.64
Primary diagnosis			
Non-Hodgkin lymphoma	Ref		
Hodgkin lymphoma	1.75	1.20–2.55	0.004
Pre-BMT radiotherapy			
No	Ref		
Yes	1.21	0.84–1.73	0.31
Income (US dollars)			
≥75,000	Ref		
50,000–74,999	3.07	1.39–6.79	0.01
<50,000	5.12	2.55–10.28	<0.0001

**Fig. 4:**
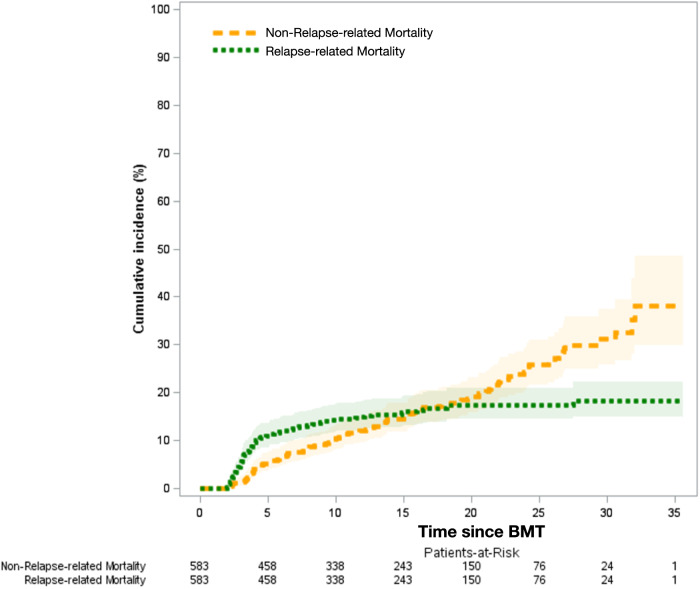
Cumulative incidence of relapse-related and non-relapse-related mortality, respectively, among 2-year survivors of autologous BMT for lymphoma, by time since BMT.

Causes of death were available for 82% of the deceased BMT cohort, and included primary disease (35%), infection (22%), subsequent malignant neoplasm (16%), cardiovascular disease (13%), pulmonary disease (4%), homicide/suicide/accident (4%), and other causes (7%). As shown in Fig. 4, relapse-related mortality (RRM) plateaued after 20 years from BMT, whereas the non-relapse-related mortality (NRM) continued to climb, crossing RRM at 20 years from BMT. The 25 years cumulative incidence of RRM was 18.2% (95% CI 14.3–20.8%), while that of NRM was 25.9% (95% CI 21.3–30.6%).

Individuals with HL were at increased risk for RRM (HR = 2.11, 95% CI = 1.31–3.42; reference: NHL) (Table 4). Other factors associated with RRM included high risk of relapse at BMT (HR = 2.21, 95% CI = 1.17–4.18; reference: standard risk), and year of transplantation (≥2000: HR = 0.37, 95% CI = 0.20–0.67; reference: <1990). Factors associated with NRM included race/ethnicity (Black individuals: HR = 3.72, 95% CI = 1.68–8.24; Hispanic individuals: HR = 1.95, 95% CI = 1.05–3.60; reference: non-Hispanic White individuals), pre-BMT radiation (HR = 2.30, 95% CI = 1.37–3.86; reference: non-irradiated) and year of transplantation (≥2000: HR = 0.30, 95% CI = 0.13–0.69; reference: <1990). When restricting the follow-up to 7 years post-BMT, treatment era was no longer significantly associated with RRM nor NRM (results not shown). The specific causes of death are presented in Supplementary Table 8; death due to cardiovascular disease was overrepresented among Black patients (17.9%) vs. non-Hispanic White patients (10.2%) or Hispanic patients (9.1%).

**Table 4:** Hazard ratio of cause-specific mortality among 2-year survivors of autologous BMT for lymphoma performed before age 40 years.

	Relapse-related mortality			Non-relapse-related mortality		
	HR	95% CI	*p* value	HR	95% CI	*p* value
Race/ethnicity						
Non-Hispanic white				Ref		
Black				3.72	1.68–8.24	0.001
Hispanic				1.95	1.05–3.60	0.03
Other				1.35	0.47–3.83	0.58
Year of BMT						
<1990	Ref			Ref		
1990–1999	0.59	0.35–1.01	0.05	0.78	0.41–1.49	0.45
≥2000	0.37	0.20–0.67	0.001	0.30	0.13–0.69	0.004
Primary diagnosis						
Non-Hodgkin lymphoma	Ref					
Hodgkin lymphoma	2.11	1.31–3.42	0.002			
Disease status at BMT						
Standard risk^a^	Ref			Ref		
High risk^a^	2.21	1.17–4.18	0.01	1.98	1.00–3.92	0.05
Pre-BMT radiotherapy						
No				Ref		
Yes				2.30	1.37–3.86	0.002

^a^Patients who underwent BMT in first or second complete remission were considered standard risk of relapse at transplantation; all other patients were considered at high risk.

## Discussion

Conditional on surviving the first 2 years after autologous BMT for lymphoma performed in childhood, adolescence or young adulthood, the BMT survivors were at a three-fold higher odds of experiencing a severe or life-threatening chronic health condition compared to a comparison cohort. At 20 years from BMT, almost half of the BMT recipients had developed a severe, life threatening, or fatal chronic health condition. Individuals between 30 and 39 years of age at BMT, those transplanted before the year 2000, recipients of pre-BMT radiation and those with HL were at a higher risk of a severe, life-threatening, or fatal condition. In this cohort, the 25 years all-cause mortality exceeded 60% for individuals with HL patients and 40% for those with NHL. NRM continued to climb with time since BMT, crossing RRM at 20 years post-BMT.

Previous studies have reported a high burden of long-term morbidity borne by survivors of autologous BMT for lymphoma [16–18]. However, these studies focused either exclusively on adults or on cohorts that consisted largely of adults. The current study shows that the BMT recipients were at a three-fold higher risk of a grade 3–4 chronic health condition when compared with a comparison cohort. Within the BMT survivor cohort, important findings included a 20 years cumulative incidence of a grade 3–5 condition of 50% among individuals with HL, and 43% among those with NHL. Notably, the cumulative incidence continued to increase throughout follow-up. Risk factors for grade 3–5 conditions included transplantation at age >30 years, and pre-BMT radiotherapy. In adult recipients of autologous BMT, older age has been identified as a risk factor for late effects [17]. It is well-known that chest-directed radiotherapy increases the risk for chronic health conditions, especially subsequent malignant neoplasms and cardiovascular and pulmonary disease [19, 20], and that TBI increases the risk for a wide range of late effects [21, 22]. In the current study, pre-BMT radiotherapy was a significant predictor of a grade 3–5 chronic health condition, while TBI was not. This was likely due to the higher dose of radiation used in the pre-BMT radiation vs. TBI (median dose: 32.5 vs. 12.0 Gy). Patients transplanted in year 2000 or later had a lower risk for grade 3–5 conditions. Notably, none of the examined risk factors explained this decline in risk.

A CIBMTR report of 2-year survivors of autologous BMT for classical HL or diffuse large B-cell lymphoma (DLBCL) between 1990 and 2008, included a sub-analysis of 606 patients transplanted between 15 and 39 years of age, showing a 5 years overall survival of 91% for classical HL and 97% for DLBCL patients [9]. These 5 years survival rates, conditional on surviving 2 years, are higher than those of the present cohort (HL: 75% and NHL: 88%). The disparity may be influenced by differences in inclusion, with the current report including all NHL and HL sub-types, as well as patients transplanted before 1990.

In the CIBMTR report, the NRM rate 5 years post-BMT was 7% and 2% among patients with classical HL and DLBCL, respectively, which is comparable to that of the present study (5.4%) [9]. However, in our report, with extended follow-up, we show a continuous increase in NRM with time from BMT, with a cumulative incidence of 26% at 25 years post-BMT. In contrast to NRM, the cumulative incidence of RRM plateaued after 20 years post-BMT. The most common causes of death in our cohort were primary disease, infection, subsequent malignant neoplasms and cardiovascular disease. The findings of the current study warrant that HL and NHL patients treated with autologous BMT receive life-long follow-up using a risk-stratified approach as recommended by the Children’s Oncology Group guidelines [23].

In the current report, individuals with HL, Black individuals and those with a lower income had an increased risk of all-cause mortality. We believe that the higher risk of all-cause mortality in individuals with HL compared to those with NHL could have stemmed from the higher risk of relapse-related mortality as well as a higher prevalence of chronic health conditions due, in part, to the higher proportion of HL patients receiving pre-BMT radiation (46.8%) when compared with NHL patients (25.8%), at higher median dose (35.5 Gy vs. 30.0 Gy). For NRM, there was no observed difference in risk by type of lymphoma, while pre-BMT radiotherapy was found to be a significant predictor. The finding of low income as a predictor of all-cause mortality is in line with that of Hong et al., who reported an association between lower socioeconomic status and worse outcome after autologous BMT for lymphoma in adults [24]. In contrast to the study by Hong et al., race/ethnicity was also a predictor for all-cause mortality in the present study, with Black patients being at increased risk. A notably high share of deaths among Black patients was due to cardiovascular disease, which is in line with that of black individuals in the general population in the United States [25]. Inequality regarding access to follow-up care may contribute to the higher late mortality risk seen in vulnerable sub-populations [26]. In contrast to the CIBMTR report [9], TBI did not significantly increase the risk of RRM or NRM in the present study. The differences in inclusion, with the CIBMTR report not being restricted to the sub-group of adolescents and young adults, could influence the differences in the findings between the two studies. However, in the current study, pre-BMT radiation was associated with an increase in risk for NRM.

The results from our study need to be interpreted in the context of its limitations. These include the lack of detailed information on sub-types of HL and NHL. An additional potential limitation is the use of self-reported data, potentially influenced by study participants’ access to care, awareness among healthcare providers of the risk of late effects, and communication of these outcomes to their patients. The retrospective design of the study may further increase recall bias of self-reported chronic health conditions, including the age of diagnosis. However, examining severe/life-threatening/fatal chronic health conditions likely mitigated this issue, since we were focusing on clinically overt complications. Further, our survey demonstrated a high level of sensitivity and specificity against medical record reviews [12]. The mortality results in the current report are based on causes of death registered on death certificates, where some degree of misclassification is inherent. Nonetheless, use of NDI provides the most comprehensive evaluation of vital status. Finally, while we observed a decline in late morbidity among patients transplanted in the more recent years, we were unable to identify the underlying cause(s), likely due to some unmeasured changes in transplant practice.

These limitations notwithstanding, the current investigation describes the burden of chronic health conditions and late mortality experience of a cohort of patients undergoing autologous BMT for lymphoma in children, adolescents and young adults, the largest of its kind to date, followed for a median of almost 10 years. Novel findings include a three-fold increase in risk for serious or life-threatening conditions compared to a comparison cohort and a steadily increasing cumulative incidence of serious, life-threatening or fatal conditions with time from BMT. Relapse was the leading cause of death; however, while RRM plateaus with time, NRM becomes the major cause of death after 20 years from transplantation. Lastly, Black and Hispanic individuals and those with lower socioeconomic status were most vulnerable. Findings of the present study provide evidence for long-term anticipatory follow-up, with focus on vulnerable sub-populations.

### Supplementary information

Supplemental tables and figures

## Data Availability

The datasets generated during and/or analyzed during the current study are available from the corresponding author on reasonable request.
