# Supplementary material for: Late morbidity and mortality after autologous blood or marrow transplantation for lymphoma in children, adolescents and young adults—a BMTSS report
Source: Leukemia. 2024 Feb 19;38(3):601–9. doi: 10.1038/s41375-024-02144-7 (PMC10912019; doi:10.1038/s41375-024-02144-7)
Supplement: Supplementary file 1 — Supplemental tables and figures [file 41375_2024_2144_MOESM1_ESM.pdf]

Figure legends

Supplemental Figure 1. Consort diagram of inclusion criteria for the study population of 2y-survivors of autologous BMT for lymphoma performed before the age of 40 years.

Supplemental Figure 2. Cumulative incidence of grade 3-5 chronic health conditions among 2y-survivors of autologous BMT for lymphoma performed before the age of 40 years, by treatment era.

Supplemental Table 1. Questions included in the BMTSS survey, the corresponding chronic health condition categories created from the responses and the scoring of these conditions

Organ/system -- Questions	Grade*
**Neurosensory**	
**Hearing**	
Have you ever been told by a doctor or other health care professional that you have, or have had…	
Problems hearing sounds, words, or language in crowds	1
Hearing loss requiring a hearing aid	3
Deafness in one or both ears not completely corrected by hearing aid	3
Complete deafness in either ear	3
**Vision**	
Have you ever been told by a doctor or other health care professional that you have, or have had…	
Cataracts, but did not require surgery	1
Glaucoma (excess pressure in eyeball)	1
Problems with double vision	2
Very dry eyes requiring eye drops or ointment	2
A detached retina or other condition of retina	2
Cataracts that required surgery	3
Legally blind in one or both eyes	3
Loss of an eye	3
**Oral**	
Have you ever been told by a doctor or other health care professional that you have, or have had…	
Stammering or stuttering	1
Abnormal sense of taste	1
Loss of taste or smell which has lasted at least 3 months	1
Problems with gums: bleeding or swelling	1
Abnormally dry mouth	1
**Neurological**	
Have you ever been told by a doctor or other health care professional that you have, or have had…	
Epilepsy not requiring medications	1
Repeated seizures, convulsions, or black outs not requiring medications	1
Problems with balance, equilibrium, or ability to reach for/manipulate objects	1

Persistent dizziness or vertigo	1
Tremors or problems with movement	1
Weakness or inability to move leg(s) or arm(s), with minimal impairment	1
Decreased sense of touch or feeling in hands, fingers, arms or legs	1
Epilepsy requiring medications	2
Repeated seizures, convulsions, or black outs requiring medications	2
Weakness or inability to move leg(s) or arm(s), moderate impairment	2
**Genitourinary**	
Have you ever been told by a doctor or other health care professional that you have, or have had…	
Kidney stones	1
Repeated kidney infections	2
Repeated bladder infections	2
Dialysis	4
Kidney transplant	4
**Musculoskeletal**	
Have you ever been told by a doctor or other health care professional that you have, or have had…	
Osteoporosis, brittle, weak or fragile bones	2
Avascular necrosis (deterioration of joint bones such as hip or shoulder joint)	2
Joint replacement	3
**Endocrine**	
Have you ever been told by a doctor or other health care professional that you have, or have had…	
An underactive thyroid gland (Hypothyroid) not requiring medication	1
An underactive thyroid gland (Hypothyroid), requiring you to be on medication	2
Thyroid nodules, not requiring surgery	2
Diabetes, requiring oral medications	2
Have you ever received injections of growth hormone (Protropin or Humatrope)	2
An overactive thyroid gland (Hyperthyroid)	2
Thyroid nodules, requiring removal of all or part of the thyroid gland	3
Diabetes, requiring you to be on insulin	3
Males: Gonadal failure, requiring you to be on testosterone	3
Females: Gonadal failure, requiring you to be on hormone replacement therapy	3

Females: Cessation or arrest of menses following HCT, prior to 40 years of age	3
**Cardiovascular**	
Have you ever been told by a doctor or other health care professional that you have, or have had…	
Irregular heartbeat or palpitations, (Arrhythmia) not requiring medication or follow-up by a doctor	1
Hypertension (high blood pressure), not requiring medication	1
Stiff or leaky heart valves	1
Congestive heart failure or cardiomyopathy (weak heart muscle), not requiring medication	2
Irregular heartbeat or palpitations, (Arrhythmia) requiring medication or follow-up by a doctor	2
Hypertension (high blood pressure), requiring medication	2
A myocardial infarction (heart attack)	3
Coronary heart disease not requiring catheterization ("heart cath") or surgery but on medication such as nitroglycerine	3
Angina pectoris (chest pain due to lack of oxygen to heart requiring medication such as nitroglycerine)	3
Congestive heart failure or cardiomyopathy (weak heart muscle), requiring medication	3
Blood clot in lung, arm, leg or pelvis	3
Heart attack, requiring catheterization ("heart cath"), angioplasty (enlarging a heart vessel using a balloon), or coronary artery bypass surgery	4
A stroke or a cerebrovascular accident	4
Heart transplant	4
**Respiratory**	
Have you ever been told by a doctor or other health care professional that you have, or have had…	
Chronic cough or shortness of breath for greater than one month	1
Lung fibrosis or "scarring" of the lung, not requiring extra oxygen	1
Emphysema, not requiring medication	1
Lung fibrosis or "scarring" of the lung, requiring extra oxygen	3
Emphysema, requiring medication	3
Lung transplant	4
**Gastrointestinal**	
Have you ever been told by a doctor or other health care professional that you have, or have had…	
Hepatitis	2
Rectal or anal fistula	2
Cirrhosis of the liver	3
Rectal or anal stricture (narrowing or scarring)	3

Surgery for intestinal obstruction	3
Liver transplant	4
**Second malignancy**	
Have you ever been told by a doctor or other health care professional that you have, or have had…	
Basal cell cancer	2
Thyroid cancer	3
Any other type of cancer	4

* Grade of chronic health conditions according to the Common Terminology Criteria for Adverse Events (CTCAE), v5.0

Supplemental Figure 1. Consort diagram of inclusion criteria for the study population 2y-survivors of autologous BMT for lymphoma performed before the age of 40y

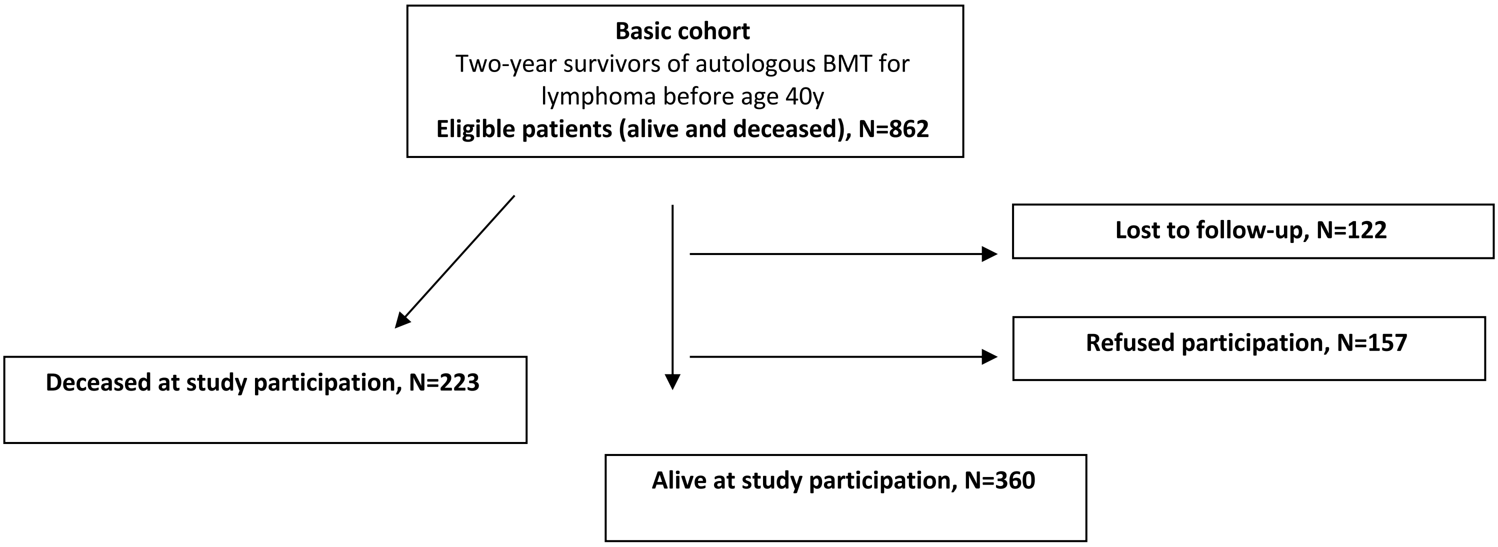

Supplemental Table 2. Demographic and clinical characteristics of participants and non-participants

	Participants N=583	Non-participants N=157	P-value
Age at BMT in years			
Median (Range)	27(5-39)	30(2-39)	0.06
Age at BMT in years, N (%)			
≤21	107(18.4%)	37(23.6%)	0.3
22-30	221(37.9%)	59(37.6%)	
>30	255(43.7%)	61(38.9%)	
Sex			
Female	272(46.7%)	50(31.8%)	0.001
Race/ethnicity			
Non-Hispanic white	424(72.7%)	98(62.4%)	0.007
Black	38(6.5%)	23(14.6%)	
Hispanic	86(14.8%)	26(16.6%)	
Other	35(6.0%)	10(6.4%)	
Year of BMT, N (%)			
<1990	76(13.0%)	2(1.3%)	<0.0001
1990-1999	251(43.1%)	36(22.9%)	
≥2000	256(43.9%)	119(75.8%)	
Primary diagnosis			
Hodgkin lymphoma	349(59.9%)	97(61.8%)	0.7
Non-Hodgkin lymphoma	234(40.1%)	60(38.2%)	
Treating Institution			
City of Hope	375(64.3%)	108(68.8%)	0.5
UMN	143(24.5%)	18(11.5%)	
UAB	65(11.1%)	31(19.7%)	

UMN=University of Minnesota, UAB=University of Alabama at Birmingham

Supplemental Table 3. Demographic characteristics of autologous BMT survivors alive at study participation and the comparison cohort

	Survivors (N=360)		Comparison cohort (N=1070)		
**Variable**	**Median**	**Range**	**Median**	**Range**	**P-value**
Age at study participation	44.0	19-67	54.7	19-67	<0.0001
	**N**	**%**	**N**	**%**	**P-value**
Sex					
Female	174	48.3	645	60.3	<0.0001
Male	186	51.7	425	39.7	
Race/ethnicity					
Non-Hispanic white	270	75.0	907	84.8	0.0001
Hispanic	53	14.7	83	7.8	
Black	10	2.8	28	2.6	
Other	27	7.5	52	4.9	
Education					
Less than high school	53	14.7	110	10.3	0.11
High school to college	126	35.0	369	34.5	
≥College	179	49.7	582	54.4	
Income (US dollars)					
<50000	101	28.1	209	19.5	0.001
50000-74999	62	17.2	183	17.1	
≥75000	158	43.9	584	54.6	
Missing	39	10.8	94	8.8	
Current Insurance					
No Insurance	16	4.4	7	0.7	<0.0001
With Insurance	344	95.6	914	85.4	
Chronic health condition					
Grade 1-2	522	48.8	164	45.6%	0.3
Grade 3-4	287	26.8%	145	40.3%	<0.001

Supplemental Table 4. Odds ratio of any grade 3-4 chronic health conditions in recipients of autologous BMT before age 40y alive at study participation compared with a comparison cohort

		Univariate analysis			Multivariate analysis		
	N (%)	OR	95% CI	p-value	OR	95% CI	p-value
Group, N (%)							
Comparison cohort	1070(74.8%)	Ref			Ref		
BMT recipients	360(25.2%)	1.80	1.40-2.31	<0.0001	3.03	2.25-4.07	<0.0001
Age at study participation (years), N (%)							
<35	236(16.5%)	Ref			Ref		
35-55	637(44.5%)	3.00	1.94-4.64	<0.0001	2.91	1.86-4.54	<0.0001
>55	557(39.0%)	5.24	3.39-8.10	<0.0001	7.69	4.85-12.20	<0.0001
Sex, N (%)							
Female	816(57.3%)	Ref					
Male	608(42.7%)	1.12	0.89-1.40	0.3			
Race/ethnicity, N (%)							
Non-Hispanic white	1177(82.3%)	Ref					
Black	38(2.7%)	1.89	0.99-3.63	0.06			
Hispanic	136(9.5%)	1.08	0.74-1.58	0.7			
Other	79(5.5%)	0.64	0.37-1.11	0.1			
Education, N (%)							
<High School	163(11.4%)	Ref					
High School/some college	495(34.6%)	0.81	0.56-1.17	0.3			
≥College	764(53.2%)	0.62	(0.44-0.89	0.01			
Missing	11(0.8%)						
Income (US dollars), N (%)							
≥75000	742(51.9%)	Ref					
<50,000	310(21.7%)	1.19	0.90-1.59	0.2			
50000-74999	245(17.1%)	1.01	0.74-1.39	0.9			
Missing	133(9.3%)						

Supplemental Figure 2

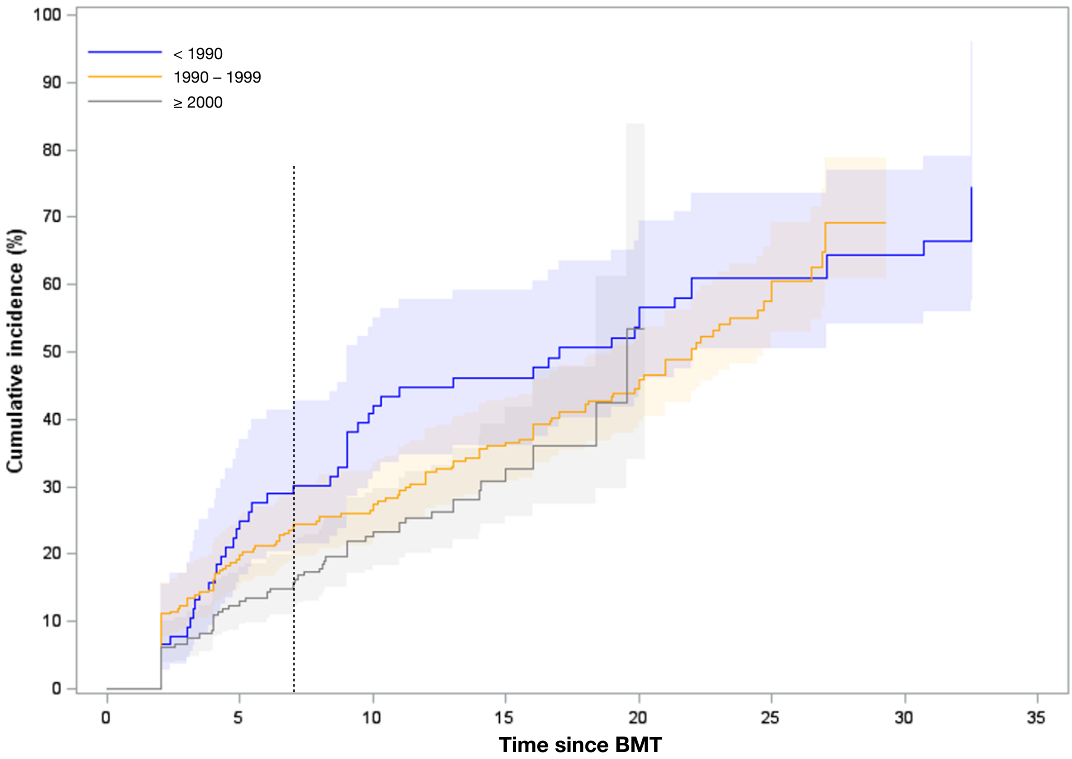

Patients-at-Risk

< 1990	76	48	31	24	18	13	7	0
1990 – 1999	252	167	131	102	53	12	0	
≥ 2000	255	192	94	26	1	0		

Supplemental Table 5. Mediation analysis of treatment era vs. any grade 3-5 condition among autologous BMT recipients for Non-Hodgkin lymphoma performed <40y

Treatment era	Mediator	HR (95% CI)	p-value
1990-1999 vs. <1990	No mediation	0.82(0.41-1.61)	0.56
>=2000 vs. <1990		0.40(0.18-0.87)	0.02
1990-1999 vs. <1990	Age at BMT	0.72(0.37-1.42)	0.35
>=2000 vs. <1990		0.36(0.17-0.79)	0.01
1990-1999 vs. <1990	Sex	0.76(0.38-1.53)	0.44
>=2000 vs. <1990		0.39(0.18-0.84)	0.02
1990-1999 vs. <1990	Race/ethnicity	0.82(0.41-1.63)	0.57
>=2000 vs. <1990		0.36(0.16-0.79)	0.01
1990-1999 vs. <1990	Stem cell source	0.76(0.39-1.49)	0.43
>=2000 vs. <1990		0.35(0.16-0.79)	0.01
1990-1999 vs. <1990	Total body irradiation	0.82(0.41-1.65)	0.57
>=2000 vs. <1990		0.40(0.19-0.85)	0.02
1990-1999 vs. <1990	Disease status at BMT	0.72(0.33-1.56)	0.40
>=2000 vs. <1990		0.36(0.15-0.87)	0.02
1990-1999 vs. <1990	PreBMT treatment Anthracyclines	0.69(0.28-1.68)	0.41
>=2000 vs. <1990		0.40(0.16-0.99)	0.05
1990-1999 vs. <1990	PreBMT treatment Antimetabolites	0.70(0.34-1.43)	0.32
>=2000 vs. <1990		0.31(0.13-0.72)	0.01
1990-1999 vs. <1990	PreBMT treatment Bleomycin	0.75(0.37-1.51)	0.42
>=2000 vs. <1990		0.33(0.14-0.75)	0.01
1990-1999 vs. <1990	PreBMT treatment Monoclonal antibodies	0.79(0.39-1.60)	0.52
>=2000 vs. <1990		0.35(0.14-0.91)	0.03
1990-1999 vs. <1990	PreBMT treatment Platinum	0.84(0.42-1.69)	0.63
>=2000 vs. <1990		0.45(0.20-1.04)	0.06
1990-1999 vs. <1990	PreBMT treatment Topoisomerase inhibitors	0.85(0.42-1.72)	0.66
>=2000 vs. <1990		0.45(0.20-1.03)	0.06
1990-1999 vs. <1990	PreBMT radiotherapy	0.81(0.40-1.63)	0.55
>=2000 vs. <1990		0.39(0.18-0.88)	0.02
1990-1999 vs. <1990	Conditioning including Carmustine	0.80(0.40-1.61)	0.54
>=2000 vs. <1990		0.41(0.19-0.87)	0.02
1990-1999 vs. <1990	Conditioning including Etoposide	0.92(0.46-1.83)	0.81
>=2000 vs. <1990		0.49(0.22-1.12)	0.09
1990-1999 vs. <1990	Conditioning including Nitrosourea	0.82(0.40-1.65)	0.57
>=2000 vs. <1990		0.40(0.19-0.85)	0.02
1990-1999 vs. <1990	Education level	0.74(0.36-1.50)	0.40
>=2000 vs. <1990		0.35(0.16-0.77)	0.01
1990-1999 vs. <1990	Income	0.75(0.37-1.49)	0.41
>=2000 vs. <1990		0.36(0.16-0.80)	0.01

Supplemental Table 6. Prevalence of grade 3-5 chronic health conditions among 583 recipients of autologous BMT performed before age 40y

Chronic health condition	All patients N=583	Hodgkin lymphoma N=349	NHL N=234	Non-Hispanic white N=424	Black N=38	Hispanic N=86	Other N=35
Any grade 3-5 chronic health condition	241(41.3%)	148(42.4%)	93(39.7%)	175(41.3%)	15(39.5%)	41(47.7%)	10(29.4%)
Any grade 3-5 condition excluding SMN	193(33.1%)	127(36.4%)	66(28.2%)	136(32.1%)	14(36.8%)	36(41.9%)	7(20.6%)
Subsequent malignant neoplasm	67(11.6%)	37(10.6%)	30(13.0%)	47(11.2%)	2(5.3%)	14(16.3%)	4(12.1%)
Cardiovascular disease*	61(10.5%)	39(11.2%)	22(9.5%)	41(9.7%)	7(18.4%)	10(11.6%)	3(8.8%)
Blood clot	19(3.3%)	10(2.9%)	9(3.9%)	16(3.8%)	0	2(2.3%)	1(2.9%)
Joint replacement	17(2.9%)	10(2.9%)	7(30.%)	14(3.3%)	1(2.6%)	2(2.3%)	0
Sensorineural disorder**	14(2.4%)	10(2.9%)	4(1.7%)	13(3.1%)	0	1(1.2%)	0
Cataract	13(2.2%)	6(1.7%)	7(3.0%)	13(3.1%)	0	0	0
Diabetes	10(1.7%)	4(1.1%)	6(2.6%)	8(1.9%)	0	2(2.3%)	0
Lung fibrosis/transplant	10(1.7%)	9(2.6%)	1(0.4%)	5(1.2%)	1(2.6%)	2(2.3%)	2(5.9%)
Gastrointestinal disease***	10(1.7%)	7(2.0%)	3(1.3%)	7(1.7%)	0	3(3.5%)	0
Thyroid nodules	6(1.0%)	3(0.9%)	3(1.3%)	4(1.0%)	0	1(1.2%)	1(2.9%)

NHL=Non-Hodgkin lymphoma; *Includes heart attack, congestive heart failure, stroke, stiff or leaky valve; **Includes hearing loss, balance/vertigo, legally blind; ***Includes liver disease, rectal disorders, intestinal obstruction

Supplemental Table 7. Ten-year cumulative incidence of grade 3-5 chronic health conditions among 583 2y-survivors of autologous BMT for lymphoma performed before age 40y

Chronic health condition	Years after BMT	Numbers of events	CIF	95%CI
Any grade 3-5 chronic health condition	5	102	17.6%	14.6%-20.8%
	10	153	27.8%	24.1%-31.6%
Any grade 3-5 chronic health condition, excluding SMN	5	85	14.9%	12.1%-17.9%
	10	127	23.6%	20.1%-27.4%
Cardiovascular disease*	5	17	3.4%	2.1%-5.1%
	10	29	6.1%	4.2%-8.5%
Second malignant neoplasm	5	17	3.0%	1.8%-4.7%
	10	28	5.6%	3.8%-7.8%
Sensorineural disorder**	5	7	1.2%	0.6%-2.4%
	10	8	1.5%	0.7%-2.8%
Gastrointestinal disease***	5	5	0.9%	0.3%-1.9%
	10	7	1.4%	0.6%-2.7%
Cataract	5	3	0.6%	0.2%-1.6%
	10	8	1.7%	8.0%-3.2%
Diabetes	5	1	0.2%	0.02%-0.9%
	10	5	1.2%	0.5%-2.8%

*Includes heart attack, congestive heart failure, stroke, stiff or leaky valve; **Includes hearing loss, balance/vertigo, legally blind; ***Includes liver disease, rectal disorders, intestinal obstruction

Supplemental Table 8. Causes of death among 2y survivors of autologous BMT for lymphoma performed <40y

		Cause of death																	
		All cause death		Primary disease		SMN		Infection		CVD		Pulmonary disease		External causes		Other causes		Unknown	
		N	%	N	%	N	%	N	%	N	%	N	%	N	%	N	%	N	%
Total number of deceased patients		271	46.5%	94	34.7%	44	16.2%	32	11.8%	29	10.7%	9	3.3%	8	3.0%	6	2.2%	49	18.1%
Sex	Female	126	46.3%	46	36.5%	21	16.7%	15	11.9%	15	11.9%	6	4.8%	1	0.8%	1	0.8%	21	16.7%
	Male	145	46.6%	48	33.1%	23	15.9%	17	11.7%	14	10.0%	3	2.1%	7	4.8%	5	3.5%	28	19.3%
Race/ethnicity	White	187	44.1%	71	38.0%	27	14.4%	23	12.3%	19	10.2%	5	2.7%	6	3.2%	4	2.1%	32	17.1%
	Hispanic	44	51.2%	15	34.1%	13	29.6%	4	9.1%	4	9.1%	1	2.3%	1	2.3%	2	4.6%	4	9.1%
	Black	28	73.7%	6	21.4%	1	3.6%	4	14.3%	5	17.9%	1	3.6%	1	3.6%	0		10	35.7%
	Other	12	34.3%	2	16.7%	3	25.0%	1	8.3%	1	8.3%	2	16.7%	0		0		3	25.0%
Primary diagnosis	HL	190	54.4%	71	37.4%	31	16.3%	22	11.6%	17	9.0%	7	3.7%	2	1.1%	5	2.6%	35	18.4%
	NHL	81	34.6%	23	28.4%	13	16.1%	10	12.4%	12	14.8%	2	2.5%	6	7.4%	1	1.2%	14	17.3%
Time since BMT, years	2-9	173	70.6%	82	47.4%	23	13.3%	19	11.0%	10	5.8%	3	1.7%	3	1.7%	5	2.9%	28	16.2%
	10-14	29	30.5%	5	17.2%	8	27.6%	7	24.1%	2	6.9%	2	6.9%	4	13.8%	0		1	3.5%
	≥15	69	28.4%	7	10.1%	13	21.7%	6	8.7%	15	21.7%	4	5.8%	1	1.5%	1	1.5%	20	29.0%

SMN=Subsequent malignant neoplasm, CVD=Cardiovascular disease, HL=Hodgkin lymphoma, NHL=Non-Hodgkin lymphoma
